# Advancing primary care: Establishing family medicine specialty in Tanzania

**DOI:** 10.4102/phcfm.v15i1.4248

**Published:** 2023-12-04

**Authors:** Eric L. Aghan, Henry Ziegler, Donatus R. Mutasingwa, Enica R. Massawe, Peter J. Wangwe, Dennis Lyakurwa, Muzdalifat Abeid, Riaz Ratansi, Nadeem Kassam, Esther Johnston

**Affiliations:** 1 Family Medicine Centre for Research and Development, Socio-Economic and Education Transformation, Dar es Salaam, Tanzania; 2 Department of Family Medicine, Aga Khan University, Dar es Salaam, Tanzania; 3 Lead Family Medicine Development (informal), Muhimbili University of Health and Allied Sciences, Dar es Salaam, Tanzania; 4 School of Public Health and Global Health, University of Washington, Seattle, United States; 5 Department of Internal Medicine, Medical College of Wisconsin, Milwaukee, United States; 6 Health Tanzania Foundation, Arlington, United States; 7 Department of Family Medicine, Oak Valley Health, Markham, Canada; 8 Department of Family and Community Medicine, Faculty of Family Medicine, University of Toronto, Toronto, Canada; 9 Department of Otorhinolaryngology, Muhimbili University of Health and Allied Sciences, Dar es Salaam, Tanzania; 10 Department of Obstetrics and Gynecology, Muhimbili University of Health and Allied Sciences, Dar es Salaam, Tanzania; 11 Department of Curative Services, Ministry of Health, Dodoma, Tanzania; 12 Department of Obstetrics and Gynecology, Aga Khan University, Dar es Salaam, Tanzania; 13 Department of Cardiology, Aga Khan University, Nairobi, Kenya; 14 Department of Family Medicine, The Wright Center National Family Medicine Residency at HealthPoint, Auburn, United States

**Keywords:** family medicine, family physician, primary health care, Tanzania, training

## Abstract

Family medicine has existed as a training pathway through a private university in Tanzania since 2004. As global calls have increased to embrace primary health care as a pathway to ensuring universal health coverage, so has Tanzania recently turned to explore family medicine as a specialty to improve access to comprehensive, high-quality healthcare for her entire population. This article outlines ongoing efforts to define competencies and skills of a family medicine physician in Tanzania, engage government support and open the first public university training programme for family medicine postgraduate education.

## Introduction

Over the last decade, emboldened by signing of the Declaration of Astana in 2018, there has been a growing affirmation of healthcare as a human right, and that strengthening primary health care is the most effective means to achieving universal healthcare.^1^ In this context, public health professionals, government leaders, and clinicians in Tanzania have come together to renew commitment to the discipline of family medicine (FM). This is seen to ensuring optimal access to promotive, preventive and curative care across lifespan, in a variety of contexts, and with the fewest number of physicians.^2^ This article describes progress towards developing a new role for FM in the Tanzanian health system and a new graduate training pathway.

## Understanding population health in Tanzania

Over 61.7 million people live in the United Republic of Tanzania, mostly in rural areas (64%), on both Tanganyika and Isle of Zanzibar.^3^

As of 2019, neonatal disorders and lower respiratory tract infections ranked as the top two causes of death. While human immunodeficiency virus and acquired immunodeficiency syndrome, tuberculosis, malaria and diarrhoeal diseases, are still listed in the top 10, they have fallen in rank over the last decade. Non-communicable conditions, such as stroke and ischaemic heart disease, have climbed as major drivers of mortality.^4^ The top five risk factors contributing to morbidity and mortality have remained almost unchanged over the last decade: malnutrition, air pollution, unsafe sex, water and sanitation, and high blood pressure.^4^

These statistics reveal deep need for clinicians at the primary care level in Tanzania who can lead efforts to reduce preventable diseases while managing both communicable and non-communicable diseases.

## The Tanzanian health system

World Health Organization (WHO) suggests an optimal mix of health workers to provide universal access to healthcare would ensure a minimum ratio of 80 medical doctors for every 100,000 people.^5^ However, the United Republic of Tanzania’s most recent statistics show the country has only 10 physicians for every 100,000 people.^5^ Although the nation has made progress towards universal health coverage (UHC) targets, it remains at a UHC effective coverage index 55.2.^4^

The Tanzania public healthcare system is arranged in tiers in a hierarchical structure. Primary care is the first entry point through village health posts and community dispensaries. The next level is rural health centres (which offer inpatient care) at the municipal ward level. These centres refer to the district level hospitals, which further refer patients to regional, zonal, specialised and ultimately national (tertiary) hospitals. Staffing at every level is defined by the Ministry of Health (MoH).^6^ Dispensaries are staffed by clinical officers (COs) and nurses, while health centres are intended for medical officers (MOs), COs and assistant medical officers (AMOs). District hospitals are staffed by MOs, supported by AMOs and COs. The service packages provided at each tier within the health system are defined in the national standard treatment guidelines. If a further treatment is indicated, then the patient is referred to the next level facility.^7^

However, while referral guidelines exist, referral pathways are not always followed. Patients may bypass lower levels and access care at a higher level. Furthermore, transfer from lower levels of care is not always easy or possible, even when a need for this exists, and there may be large geographic distances between health facilities with long transit times.^8,9^ Inadequate training of primary care providers and reliance on less trained cadres of healthcare workers create a mismatch between the desired service package and the ability to provide this. This may also increase the referral of patients to the next level of expertise. Previous studies have identified poor pre-transfer care and late referrals, as major contributors to tertiary hospital mortality rates.^9,10,11,12^

The government of Tanzania has invested heavily in non-physician clinicians to staff primary care facilities.^6^ However, given the enormous challenges of ensuring high quality of care for a double burden of non-communicable and communicable diseases, to a heavily rural population in a geographically large area, the government has recently begun to re-examine FM as a discipline, which may better serve the complexity and range of health needs throughout the district level.^13^

## The history of family medicine in Tanzania

Aga Khan University (AKU) Tanzania began to offer Master of Medicine in FM in 2004, after receiving approval by Tanzania Commission for Universities (TCUs). The programme is based in a private Aga Khan Hospital system and thus graduates are best trained for urban micro-health system. Only 18 graduates have completed the training thus far. Informal surveys have shown that they are mainly employed in teaching institutions in Kenya and the AKU Dar es Salaam. Remaining graduates are absorbed by the private sector and by non-governmental organisations, where they are in high demand. Historically, graduates have not found employment in public health system.

In 2014, the Medical Council of Tanganyika (MCT) first recognised FM graduates as medical specialists, a milestone in allowing graduates to obtain specialist recognition and commensurate pay. However, because of limited funding and deep historical investment and reliance on COs and AMOs for generalist care provision, FM training and absorption through government health facilities has not been realised.

## Renewing a national push for family medicine

Tanzania’s demographics and population profile highlight the need for a medical specialist, capable of serving as a clinician to people of all ages, providing both preventive and curative care for infectious and non-communicable diseases in a variety of rural and urban settings. Over the last decade, public health professionals, clinicians and other health leaders from Tanzania and around the globe have congregated to deliberate on how to best reinvest and expand FM training and practice to meet this need.

In 2014, Health Tanzania Foundation, an American based non-governmental organisation (NGO), in partnership with AKU, regional religious leaders and academic heads, established an interfaith partnership. This partnership established; Social Economic Education Transformation (SEET) as an NGO in 2016.^14^ The SEET unites academic institutions, community groups, churches and mosques within the urban underserved communities of Buguruni and Vingunguti in Dar es Salaam, as well as the rural Rufiji District, to provide training around wellness and violence prevention. The SEET has been a driving force in recognising Tanzania’s need for a fit-for-purpose family physician (FP) trained at the district level.

**FIGURE 1:**
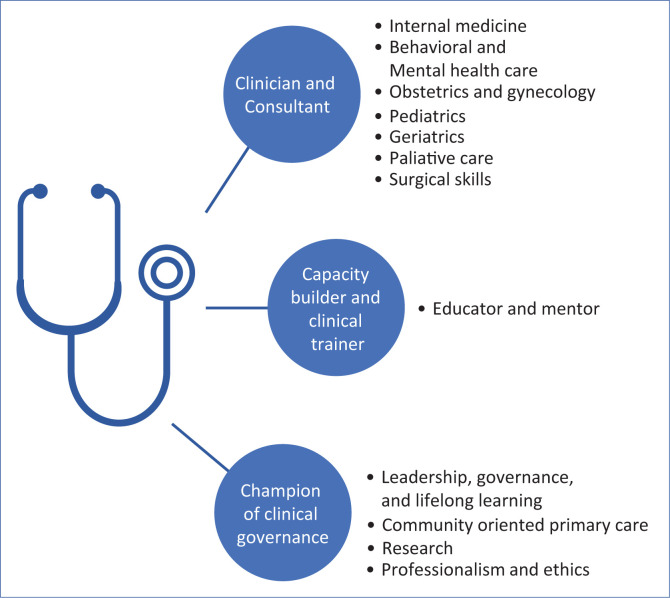
The competencies and skill sets of a Tanzanian family physician.

In 2021, with growing recognition of the need for family physician, the AKU hosted the first ever international conference to define competencies for Tanzanian family physician. They were guided by recommendations from the World Organization of Family Doctors (WONCA) and expert guidance from both national and international colleagues (see Figure 1).

Subsequently in 2021, SEET joined hands with the AKU, Muhimbili University of Health and Allied Sciences (MUHAS) and Kairuki University to establish the Family Medicine Development and Research Centre (FMDRC). This united; universities, non-governmental partners and community members who have the aim of developing community-oriented FM for Tanzania. This group envisioned a family physician who would be trained at the district level, improving the likelihood of retention in underserved rural and urban areas.^15^

Over the next few years, this reinvigorated academic, private institutions and NGO partnership to conduct outreach efforts to governmental leaders to make the case for FM. In 2022, representatives of the FMDRC presented the roles of a FM specialist to the National Health Insurance Fund (NHIF) and to the Minister for Health. As conversations developed, it became clear to all involved that investment in FM might be able to secure cost savings for a health system determined to ensure access to affordable and quality primary care for all Tanzanians.

## Envisioning family medicine’s future in Tanzania: Launching a public university training pathway

As planning for FM’s expansion continues, the need for additional training sites to meet the national needs for FM specialists has become clear. Muhimbili University of Health and Allied Sciences has stepped forward to join these efforts, alongside other public and private universities.

Aiming to develop graduates with the competencies identified in 2021 at the first national conference on FM, the new Master of Medicine (MMed) in FM programme will recruit physicians who have completed their undergraduate medical training as well as 1 year of internship, which is mandatory for registration as an independent practitioner by the MCT.

Training for the MUHAS postgraduate FM residents, the first to occur through a public university, will involve clinical rotations in an urban faith-based community health centre, as well as at rural Kisarawe district hospital, located approximately one and half hours from MUHAS, on the outskirts of Dar es Salaam.

Given the distance, from the specialist-driven care at Muhimbili National Hospital in central Dar es Salaam, trainees are offered an opportunity to develop a broad breadth of clinical skills for both differentiated and undifferentiated problems. With strong internet connectivity available at both sites, the training programme is planned to employ information technology (IT) and teaching innovations to deliver the curriculum in a blended manner. It is expected that IT application will accord the trainees strong connections with MUHAS for both digital or virtual learning materials and supportive supervision.

The curriculum for the new programme was reviewed by key stakeholders, in January and March 2023 meetings. The stakeholders were drawn from the MoH, University professionals and senior leadership, pending final approval.

## Next steps

Launching a new training programme, particularly in the public setting, requires funding. Near-term efforts to officially launch the new MMed in FM degree will depend on the acquisition of seed financing to hire programme staff and obtain initial training materials. In the long term, the programme’s sustainability will be dependent on integration into the typical funding cycles of the public university.

Additional efforts to ensure a career pathway for the programme’s graduates continue. The programme is most likely to be successful in recruiting, training and retaining FM specialists in the public health system if the ministry can identify placement targets and posts for the graduates at the district level.

## Conclusions

Tanzania has turned to FM as a solution to ensuring high-quality preventive care and addressing a double burden of infectious and non-communicable diseases for a majority rural population over a broad geographical area. Although a graduate training pathway has existed in the country since 2004, the lack of a public sector training pathway has the limited uptake of graduates into the public health system. In 2014, the MCT first recognised FM graduates as specialists and in 2021 a broad swath of Tanzanian and global consultants came together to define competencies and skill sets for Tanzanian family physicians. The country is now looking forward to open its first graduate FM training programme to train family physicians within the public sector. Although there is still much to be performed to ensure adequate integration and uptake of this programme’s graduates, the progress made thus far has ensured that the time is now optimal to establish FM in Tanzania.
